# Tropical Protozoan Diseases: Natural Product Drug Discovery and Development

**DOI:** 10.1155/2013/404250

**Published:** 2013-11-17

**Authors:** Liliana Muschietti, Roser Vila, Valdir Cechinel Filho, William Setzer

**Affiliations:** ^1^Department of Pharmacognosy, Faculty of Pharmacy and Biochemistry, University of Buenos Aires, 1113 Buenos Aires, Argentina; ^2^Unitat de Farmacologia i Farmacognòsia, Facultat de Farmàcia, Universitat de Barcelona, 08028 Barcelona, Spain; ^3^Universidade do Vale do Itajaí, Núcleo De Investigações Químico-Farmacêuticas (NIQFAR), CCS, Brazil; ^4^Department of Chemistry, University of Alabama in Huntsville, Huntsville, AL 35899, USA

Infections caused by protozoan parasites such as Chagas disease, human African trypanosomiasis, leishmaniasis, or malaria are responsible for considerable morbidity and mortality worldwide with devastating social and economic consequences (Table 1) [1]. Under normal circumstances (efficient epidemiological surveillance programs and sanitary education) the control of these diseases can be carried out effectively. Nevertheless, the implementation of an adequate health care system to palliate the necessities of the affected populations is hindered by the lack of financial and human resources, political instability in these countries, and often questionable government prioritization [2].

Currently, there is lack of effective, safe, and affordable therapies for the treatment of these diseases. The drugs used are far from ideal, and many of them were introduced decades ago; thus the development of new and more effective drugs with fewer side effects represents a crucial dare. 

Over the last century, natural products have provided molecules with high structural diversity and “drug-like” properties from a physicochemical point of view. The reason resides in their chemical and steric complexity since they have well-defined three-dimensional structures, improved in terms of efficiency and selectivity for the molecular target [3, 4]. Natural product research has made a significant contribution to the chemotherapy of parasitic diseases such as quinine and artemisinin whose analogs are currently in use for the treatment of malaria.

This special issue brings significant works, done by leading scientists, and provides an overview on and an insight into recent advances that will contribute to the discovery of natural compounds with high potential against these protozoan parasites. 

The readers will find, in nine papers, not only a wide range of topics including the identification of natural compounds with *in vitro* and *in vivo* activity against *Trypanosoma cruzi, Plasmodium falciparum,* or *Leishmania* spp. but also recent advances in the mechanism of action of bioactive compounds and the design of semisynthetic derivatives as new more effective chemotherapeutic agents with less toxicity.

## Figures and Tables

**Table 1:** Protozoan infectious diseases.

Parasites	Disease	Occurrence	Mortality	DALYs
*Plasmodium* spp.	Malaria	219 million new cases*	660,000 deaths*	33,976,000
*Leishmania* spp.	Leishmaniasis	300,000 new cases of VL each year	Approx. 40,000 deaths due to VL	1,974,000**
		1 million new cases of CL each year		
*Trypanosoma* spp.	Human African trypanosomiasis	20,000 actual cases		1,673,000**
	Chagas disease	Approx. 8 million cases***	12,000	430,000**

VL: visceral leishmaniasis, CL: cutaneous leishmaniasis, DALY's: Disability Adjusted Life Years.

*In 2010, according to the World Malaria Report 2012. **The Global Burden of Disease Report, WHO, 2004. ***Second WHO Report on Neglected Tropical Diseases 2013.
